# Supplementary figures and images for: p166 links membrane and intramitochondrial modules of the trypanosomal tripartite attachment complex
Source: PLoS Pathog. 2022 Jun 16;18(6):e1010207. doi: 10.1371/journal.ppat.1010207 (PMC9242489; doi:10.1371/journal.ppat.1010207)

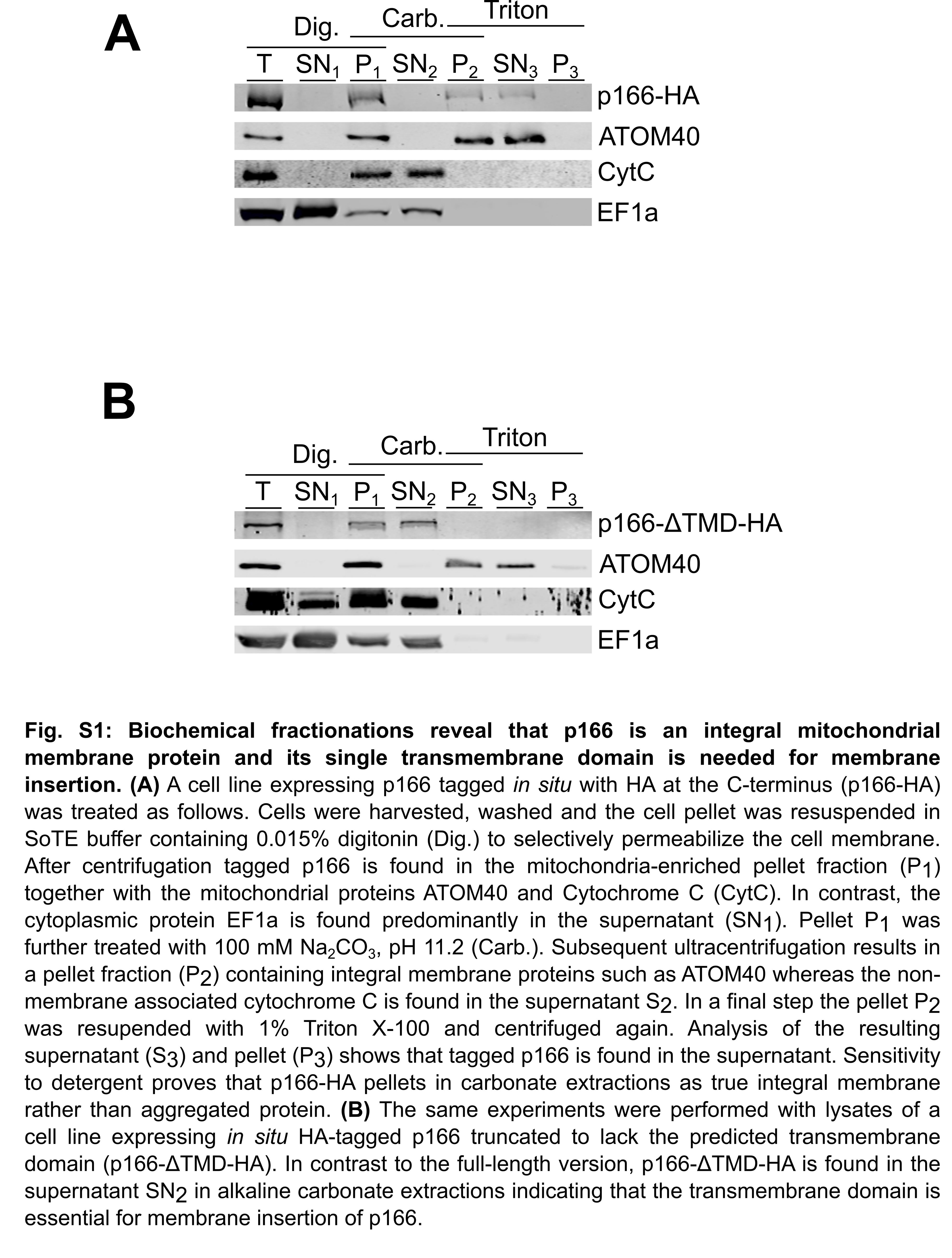

Supplement: S1 Fig — (A) A cell line expressing p166 tagged in situ with HA at the C-terminus (p166-HA) was treated as follows. Cells were harvested, washed and the cell pellet was resuspended in SoTE buffer containing 0.015% digitonin (Dig.) to selectively permeabilize the cell membrane. After centrifugation tagged p166 is found in the mitochondria-enriched pellet fraction (P1) together with the mitochondrial proteins ATOM40 and Cytochrome C (CytC). In contrast, the cytoplasmic protein EF1a is found predominantly in the supernatant (SN1). Pellet P1 was further treated with 100 mM Na2CO3, pH 11.2 (Carb.). Subsequent ultracentrifugation results in a pellet fraction (P2) containing integral membrane proteins such as ATOM40 whereas the non-membrane associated cytochrome C is found in the supernatant S2. In a final step the pellet P2 was resupended with 1% Triton X-100 and centrifuged again. Analysis of the resulting supernatant (S3) and pellet (P3) shows that tagged p166 is found in the supernatant. Sensitivity to detergent proves that p166-HA pellets in carbonate extractions as true integral membrane rather than aggregated protein. (B) The same experiments were performed with lysates of a cell line expressing in situ HA-tagged p166 truncated to lack the predicted transmembrane domain (p166-ΔTMD-HA). In contrast to the full-length version, p166-ΔTMD-HA is found in the supernatant SN2 in alkaline carbonate extractions indicating that the transmembrane domain is essential for membrane insertion of p166. (TIF) [file ppat.1010207.s001.tif]

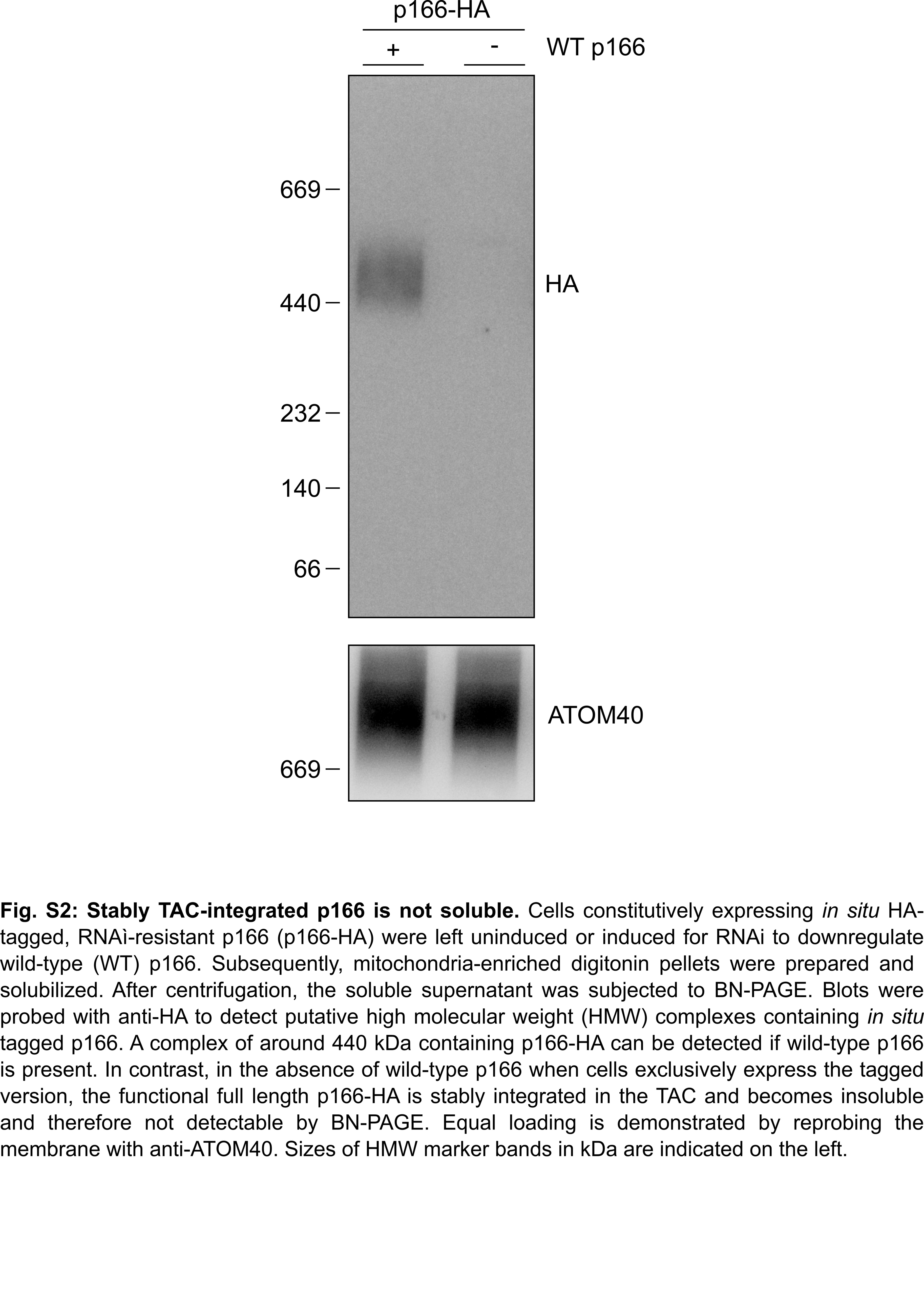

Supplement: S2 Fig — Cells constitutively expressing in situ HA-tagged, RNAi-resistant p166 (p166-HA) were left uninduced or induced for RNAi to downregulate wild-type (WT) p166. Subsequently, mitochondria-enriched digitonin pellets were prepared and solubilized. After centrifugation, the soluble supernatant was subjected to BN-PAGE. Blots were probed with anti-HA to detect putative high molecular weight (HMW) complexes containing in situ tagged p166. A complex of around 440 kDa containing p166-HA can be detected if wild-type p166 is present. In contrast, in the absence of wild-type p166 when cells exclusively express the tagged version, the functional full length p166-HA is stably integrated in the TAC and becomes insoluble and therefore not detectable by BN-PAGE. Equal loading is demonstrated by reprobing the membrane with anti-ATOM40. Sizes of HMW marker bands in kDa are indicated on the left. (TIF) [file ppat.1010207.s002.tif]

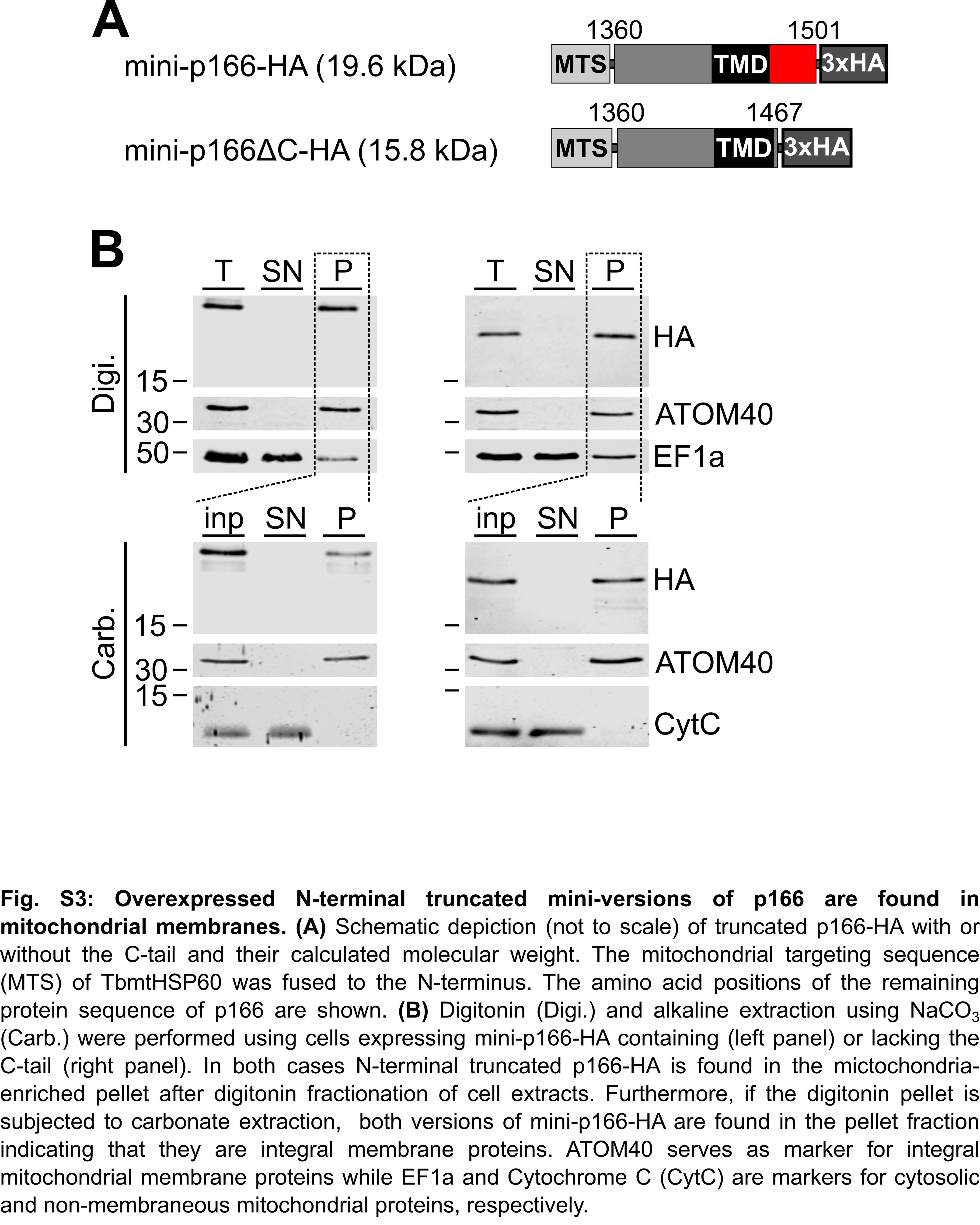

Supplement: S3 Fig — (A) Schematic depiction (not to scale) of truncated p166-HA with or without the C-tail and their calculated molecular weight. The mitochondrial targeting sequence (MTS) of TbmtHSP60 was fused to the N-terminus. The amino acid positions of the remaining protein sequence of p166 are shown. (B) Digitonin (Digi.) and alkaline extraction using NaCO3 (Carb.) were performed using cells expressing mini-p166-HA containing (left panel) or lacking the C-tail (right panel). In both cases N-terminal truncated p166-HA is found in the mictochondria-enriched pellet after digitonin fractionation of cell extracts. Furthermore, if the digitonin pellet is subjected to carbonate extraction, both versions of mini-p166-HA are found in the pellet fraction indicating that they are integral membrane proteins. ATOM40 serves as marker for integral mitochondrial membrane proteins while EF1a and Cytochrome C (CytC) are markers for cytosolic and non-membraneous mitochondrial proteins, respectively. (TIF) [file ppat.1010207.s003.tif]

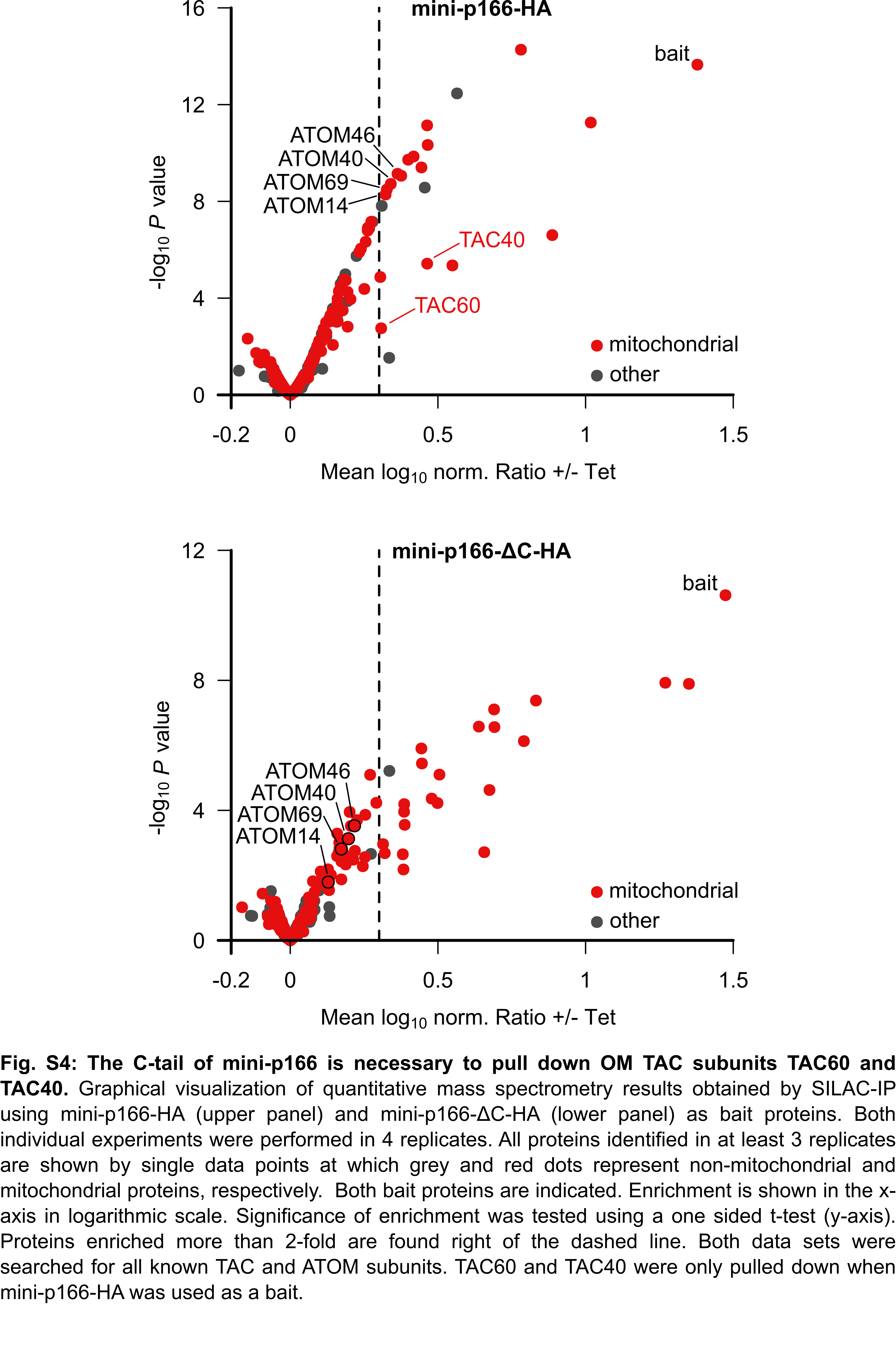

Supplement: S4 Fig — Graphical visualization of quantitative mass spectrometry results obtained by SILAC-IP using mini-p-166-HA (upper panel) and mini-p166- ΔC-HA (lower panel) as bait proteins. Both individual experiments were performed in 4 replicates. All proteins identified in at least 3 replicates are shown by single data points at which grey and red dots represent non-mitochondrial and mitochondrial proteins, respectively. Both bait proteins are indicated. Enrichment is shown on the x-axis in logarithmic scale. Significance of enrichment was tested using a one sided t-test (y-axis). Proteins enriched more than 2-fold are found right of the dashed line. Both data sets were searched for all known TAC and ATOM subunits. TAC60 and TAC40 were only pulled down when mini-p166 was used as a bait. (TIF) [file ppat.1010207.s004.tif]

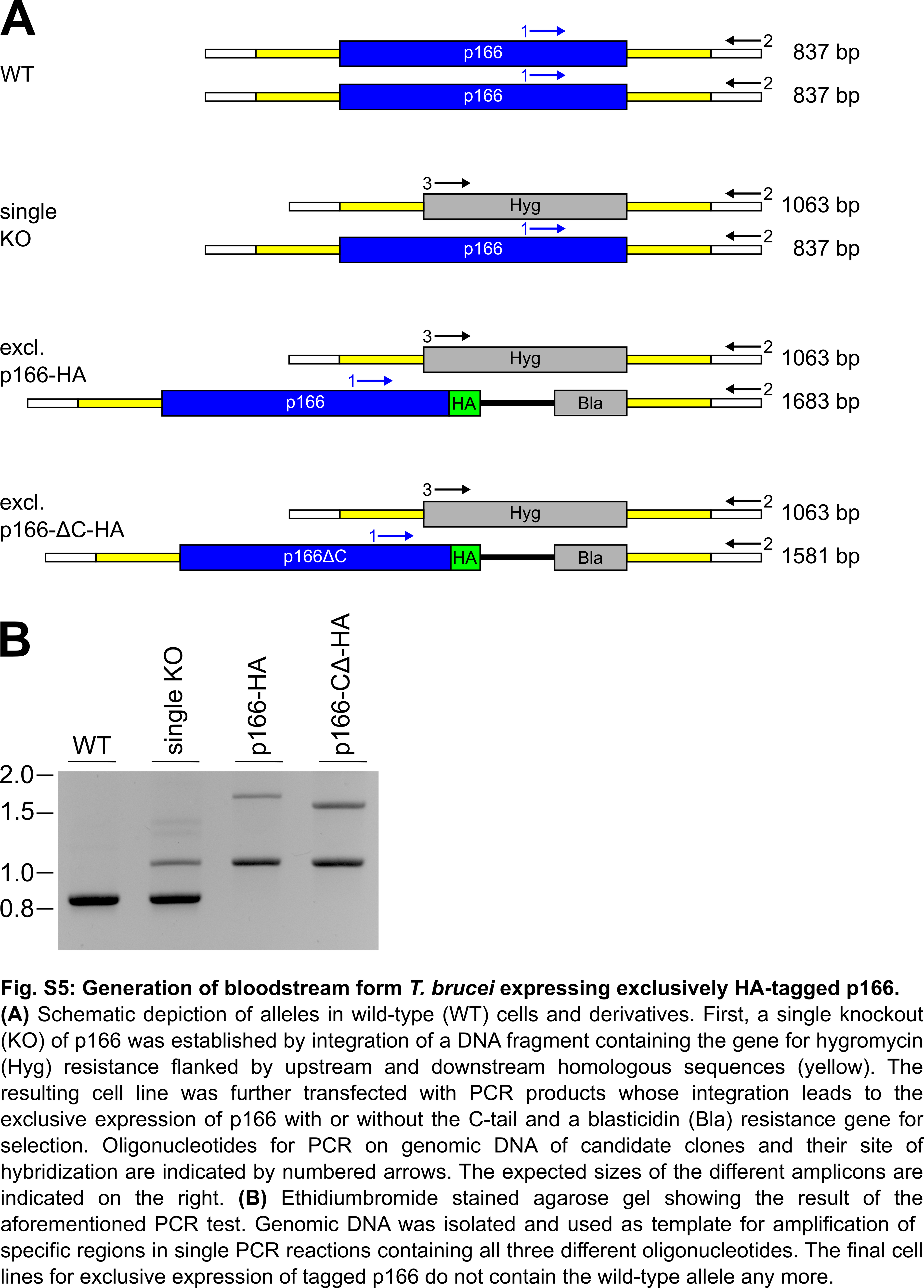

Supplement: S5 Fig — (A) Schematic depiction of alleles in wild-type (WT) cells and derivatives. First, a single knockout (KO) of p166 was established by integration of a DNA fragment containing the gene for hygromycin (Hyg) resistance flanked by upstream and downstream homologous sequences (yellow). The resulting cell line was further transfected with PCR products whose integration leads to the exclusive expression of p166 with or without the C-tail and a blasticidin (Bla) resistance gene for selection. Oligonucleotides for PCR on genomic DNA of candidate clones and their site of hybridization are indicated by numbered arrows. The expected sizes of the different amplicons are indicated on the right. (B) Ethidiumbromide stained agarose gel showing the result of the aforementioned PCR test. Genomic DNA was isolated and used as template for amplification of specific regions in single PCR reactions containing all three different oligonucleotides. The final cell lines for exclusive expression of tagged p166 do not contain the wild-type allele anymore. (TIF) [file ppat.1010207.s005.tif]
