# Supplementary material for: p166 links membrane and intramitochondrial modules of the trypanosomal tripartite attachment complex
Source: PLoS Pathog. 2022 Jun 16;18(6):e1010207. doi: 10.1371/journal.ppat.1010207 (PMC9242489; doi:10.1371/journal.ppat.1010207)
Supplement: Attachment — Submitted filename: p166 rebuttal letter-final.docx [file ppat.1010207.s007.docx]

***Reviewer's Responses to Questions***

***Part I - Summary*** *Please use this section to discuss strengths/weaknesses of study, novelty/significance, general execution and scholarship.*

*Reviewer #1: The mitochondrial genome of the parasite Trypanosoma brucei is connected to a complicated membrane structure, referred to as the tripartite attachment complex (TAC). The TAC spans through both mitochondrial membranes, thereby forming a bridge from the cytosol to the mitochondrial matrix. Several components of the TAC were described, however, the molecular architecture is still not entirely clear.
In this study, the authors characterize the TAC protein p166 and identify it as an inner membrane protein with a huge matrix domain and a short C-tail exposed to the intermembrane space (IMS). Depletion of p166 leads to the loss of mitochondrial genomes. The matrix protein TAC102 seems to be critical for mtDNA binding, in consistence with observations from the Ochsenreiter lab. Schimanski et al show that the short IMS domain of p166 makes the contact to the outer membrane protein TAC60, an interaction which even can be recapitulated in yeast, an organism which lacks a TAC equivalent.
This is an interesting study which identifies a central component in the mtDNA anchor complex of trypanosomes. The data are of high technical quality and well controlled. The topology of p166 is not unexpected given the N-terminal MTS and a C-terminal transmembrane domain. Both features were already recognized in the Zhao et al study (EMBO J, 2007) in which p166 was initially described. Nevertheless, the role of the C-terminal region remained unclear and it was speculated that the C-terminus of p166 interacts with the cytoskeleton directly. Thus, this paper now clarifies this critical point and serves as good basis to study the molecular structure and function of the TAC in more depth.*

*Reviewer #2: Reviewer’s comments PPATHOGENS-D-21-02538

In this manuscript, Schimanski et al., investigated the architecture of the tripartite attachment complex (TAC) that connects the flagellar basal body to the single mitochondrial genome known as kinetoplast (kDNA) through the double membrane of the mitochondrion in T. brucei. TAC is crucial for mitochondrial genome segregation during cell division.

Strength: Authors demonstrated that the C-terminal tail of p166, a previously identified TAC component, interact with the loop region of an outer membrane protein, TAC60, thus acts as a linker of the outer and inner module of the TAC. Although this interaction is not crucial for localization of p166 in the TAC, it is essential for k-DNA segregation. Most of the experiments are well done that provided important information regarding TAC.

Weaknesses: Some of the results are overinterpreted. Details comments are given below.

1. Previous studies showed that the full length p166 can be solubilized in 1% digitonin containing buffer and was analyzed by BN-PAGE (Hoffmann et al., PNAS 2018). Therefore, it is not clear why it is necessary to truncate a large N-terminal domain just to solubilize this protein.*

We have added an additional Figure (Fig S2) which shows that C-terminally HA-tagged full length p166 is soluble in digitonin only when the untagged wildtype p166 is present, but completely insoluble when p166-HA is expressed in an induced p166-3’UTR RNAi cell line which lacks wildtype p166 (see new Fig. S2). The reason for this is that in the presence of wildtype p166 the full length tagged version of p166 is not fully integrated into the TAC and therefore remains soluble. In contrast, if the tagged version is exclusively expressed, and therefore must take over the function from the wildtype version, it is integrated into the TAC and becomes insoluble.

The version analyzed in the Hoffmann et al. paper contained a large C-terminal PTP tag and was expressed in the presence of wild-type p166, thus it is expected to be non-functional, not fully integrated into the TAC and therefore digitonin soluble. The non-functional mini-versions of p166 on the other hand are non-functional and soluble in either the presence or the absence of wildtype full length p166. Moreover, due to their much smaller size they are less prone to proteolysis and are more easy to handle.

We have slightly modified the text in question: see “page 7, second phrase” and “page 8, third line of the third paragraph”.

*2. The N-terminal truncated version of p166 distributed all over the mitochondria, which indicates that this region is critical for proper localization of this protein at the TAC. However, authors ignored this point till at the end of discussion. Instead, they mentioned (Page 7, 3rd paragraph) “mini-p166-HA with an intact C-terminus does not depend on the large matrix-exposed N-terminus of p166 for its localization”.*

This is a misunderstanding: While the N-terminal truncated p166 (mini-p166-HA) version is indeed localized all over the mitochondria, it is still concentrated in the TAC region, which is best illustrated by the fact that it is recovered in 48% of all isolated flagella. Thus, the general mitochondrial localization that is also observed is likely caused by the overexpression of mini-p166-HA. Intriguingly, the same is not the case for the mini-p166-ΔHA version: it is not concentrated around the TAC region and only recovered in 2.8% of all isolated flagella. Thus, the statement that “mini-p166-HA with an intact C-terminus does not depend on the large matrix-exposed N-terminus of p166 for its localization” is correct.

We have slightly modified the text to better explain this issue (page 7, third paragraph), it now reads: “When whole cells were analyzed by immunofluorescence microscopy, both mini-versions of p166 were found distributed all over the mitochondrial membrane. The general mitochondrial staining is most likely caused by overexpression of the mini versions since the signals are not evenly distributed and local foci of higher signal strength could be observed some of which appear to be in close vicinity to the kDNA in the case of mini-p166-HA (Fig. 4D).”

*3. The legend of Fig. 4: This figure is not showing that the C-tail of p166 is in the IMS. Please modify the title of the figure legend*

We have now done a protease protection assay with mitoplasts which experimentally shows that the C-tail is exposed to the IMS in new Fig. 4G of the revised manuscript. Thus, we believe we can stay with the original title of the Figure legend. (For a detailed answer see Part II – Major Issues: Key Experiments Required for Acceptance, Reviewer 2, point 2)

*4. There is no evidence that the predicted TMD of p166 is responsible for membrane integration. Therefore, it is better to use the term ‘predicted’ throughout the manuscript.*

In the revised manuscript we now show that a p166 version that lacks the TMD was exclusively recovered in the supernatant in a carbonate extraction (see new Fig. S1B). This provides evidence that the predicted TMD is really a TMD (for a detailed answer see Part II – Major Issues: Key Experiments Required for Acceptance, Reviewer 2, point 1).

*5. Fig. 5B & C: there are multiple bands in the blots probed with anti-HA and anti-myc. Authors need to specify the non-specific bands*

The anti-HA and anti-myc antibodies are specific. There are no unspecific bands. However, depending on the percentages of the SDS gels used we often see clusters of bands with very similar molecular weights for different TAC subunits. They likely reflect as yet unknown posttranslational modifications. Alternatively or additionally they could be caused by limited N-terminal proteolysis.

*6. Zhou et al convincingly showed by immunogold EM that p166 is localized in the matrix side of the TAC (Zhao et al., EMBO J 2008). Therefore, the comment in the first paragraph of the discussion; “it remained unclear where exactly within the three TAC regions p166 needs to be placed” is not true. Authors need to modify this sentence.*

Zhao et al. is a fantastic study that pioneered the whole TAC field. However, in our opinion it did not exactly place p166 in respect to the three TAC subdomains. It was not shown experimentally that p166 is indeed an integral membrane protein (also because the C-terminal TMD is not essential for TAC localization of p166). Moreover, in the very nice immunogold labeling experiment the mitochondrial membranes cannot be discerned, it is therefore not possible to decide where exactly the matrix would be and whether p166 belongs to the “differentiated membrane”, to the “unilateral filaments” or to both subregions, as we now show in the present study.

*7. P166 is most likely integrated in the IM exposing the smaller C-terminal tail in the IMS, however it is not experimentally verified. Which part of the protein is protected when mitoplast is treated with proteinase K?*

As described above we have now done a protease protection assay with mitoplasts which experimentally shows that the C-tail is exposed to the IMS in new Fig. 4G of the revised manuscript. (For a detailed answer see Part II – Major Issues: Key Experiments Required for Acceptance, Reviewer 2, point 2)

*8. Authors commented that P166 is the only IM protein of the TAC, which in contrast to the multiple components of the OM. This is true so far. However, other accessory proteins in the IM could be needed for TAC formation.*

We agree with the reviewer and we apologize for this overinterpretation of the data. - What we wanted to say is that with all the components known and with the information of how they interact with each other the distance between the outer OM and the kDNA proximal TAC102 can in principle be bridged without gaps. However, this does of course not exclude that further essential TAC subunits may exist. We modified the abstract and the discussion accordingly. (For a detailed answer see Part II – Major Issues: Key Experiments Required for Acceptance; Reviewer 2, point 3).

*Particularly when p166 is found in a 700 kDa complex (Hoffmann et al., PNAS 2018). What other proteins are present in this complex? Why p166 and TAC60 are not present in the same protein complex?*

As discussed above, the 700 kDa complex in the Hoffmann et al. paper contained a large C-terminal PTP tag and was expressed in the presence of wild-type p166, thus it is likely non-functional, not integrated into the TAC and therefore digitonin soluble (see also new Fig. S2).

Detergent soluble TAC complexes that can be resolved on BN-PAGE likely represent assembly intermediates since the fully functional TAC structure is not soluble in non-ionic detergents. Moreover, there is some evidence that TAC60, TAC40 and TAC42 form a distinct subcomplex in the OM that might also be an assembly intermediate (1). Thus, there are likely distinct assembly pathways for the OM and IM TAC subregions, respectively, which could explain why TAC60 and p166 are not present in the same subcomplex.

*9. Some sentences in the abstract are over simplified. 1) “Surprisingly, non-functional p166 lacking the C-terminal 34 aa still localizes to the TAC region. This suggests the existence of non-essential TAC-associated proteins in the OM.” These non-essential TAC-associated proteins could be the filament-associated proteins or IM proteins, not only the OM-proteins.*

The reviewer is correct we cannot know where exactly these postulated TAC-associated proteins would be localized. Moreover, we can also not know whether these proteins are essential or not. Thus, we have also removed the word “non-essential”. We have modified the sentence in the abstract accordingly. It now reads: “This suggests the existence of additional TAC-associated proteins which loosely bind to non-functional p166 lacking the C-terminal 34 aa and keep it at the TAC.”

*2) “These proteins can loosely bind to non-functional p166 lacking the C-terminal 34 aa and keep it at the TAC but their binding would not be strong enough to withstand the mechanical force upon kDNA segregation.” This sentence requires modification. Due to lack of the connection between TAC60 and the C-tail of p166, kDNA was not segregated and stays attached to the old TAC, which indicates that without this interaction new TAC module is not properly formed and lose connection with the extended k-DNA. It is not clear what authors meant by mechanical force, which is undefined.*

We agree that this should be better explained. What we mean is, that the interaction of p166-ΔC with putative TAC-associated protein(s) alone, while capable of keeping p166-ΔC at the TAC, would in the context of the full length p166, not be strong enough the bear the load imposed on the TAC during the segregation of the basal bodies. We have modified the sentence in the abstract accordingly. It now reads: “However, binding of full length p166 to these TAC-associated proteins alone would not be sufficient to withstand the mechanical load imposed by the segregating basal bodies”.

*Reviewer #3: The manuscript by Schimansk et al. described a detailed analysis of p166, the first TAC component identified back in 2008. Based on current and previous studies, the authors proposed a model where p166 is a mitochondrial inner membrane (IM) protein, with the bulk N-terminal domain in the matrix and the C-terminal 34 amino acids interacting with TAC60 on the outer membrane (OM). While the C-terminal domain is not required for p166 localization to the TAC, it is essential for the normal functions of p166. The study identified p166 as an IM anchor that connects to the OM module to the kinetoplast. The interaction of p166 with other TAC components, particularly TAC60, is well established. The conclusion that p166 is the only essential IM subunit of TAC would require additional experimental support.*

We agree with the reviewer and we apologize for this overinterpretation of the data. - What we wanted to say is that with all the components known and with the information of how they interact with each other the distance between the outer membrane and the kDNA proximal TAC102 can in principle be bridged without gaps. However, this does of course not exclude that further essential and possibly also IM TAC subunits may exist. (For a detailed answer see Part II – Major Issues: Key Experiments Required for Acceptance, Reviewer 3, point 3)

*Reviewer #4: Pathogenic Kinetoplastids condense their mitochondrial genomes known as kinetoplast DNA or kDNA and physically connect them to the basal bodies of their flagella in order to obtain robust , reliable mitochondrial genome segregation. Subsequently, kDNA segregation occurs with and by the flagella during cell growth.

This manuscript addresses components in the kDNA basal body linkages and is a well written and well executed body of work. The authors identify p166 as the connective protein that traverses the inner mitochondrial membrane and places its C-terminus in the intra-membrane space. This allows p166 to interact with TAC60 and ultimately produces a system that links outer membrane proteins to the kDNA. They show that the C-ter of p166 is essential for proper TAC function and does not require kDNA for correct localisation. p166 is therefore the missing link in the kDNA-cytoplasm connection.*

*Reviewer #5: In this work, the authors describe the localization and interactions of p166, which was the first protein component of the TAC to be described. The data show that, as predicted, p166 is an integral membrane protein. They show that it has an N-terminal mitochondrial targeting signal and that it is part of the inner mitochondrial membrane. They show that the small C-terminus of p166 projects into the intermembrane space where it interacts with known TAC components. Interestingly, while the C-terminus of p166 appears to be critical for the function of the protein, it is mostly dispensable for its correct localization in the mitochondrial membrane, implying that the N-terminal region, found in the mitochondrial matrix, is involved in localization. Since a C-terminal truncated p166 still localizes to the correct region in the mitochondrion, there must be as yet undiscovered protein factors that interact with p166 and localize it to the TAC region.

This paper makes an important contribution in that, after so much activity in recent years to identify more TAC components and determine their localization, interactions, and order of assembly, it’s nice to see p166, the first TAC component, be integrated into this model, particularly in a way that opens up new questions for research in the field. It’s clear there are many aspects of this fascinating structure that are yet to be revealed, and this work stands as a very thorough investigation of this critical component. The experiments are convincing, careful, and well-presented.*

***Part II – Major Issues: Key Experiments Required for Acceptance*** *Please use this section to detail the key new experiments or modifications of existing experiments that should be absolutely required to validate study conclusions.

Reviewer #1: 1. The topology of p166 in the inner membrane is well documented in this study. However, the characterization of the matrix domain is very superficial and the statements to the model shown in Fig. 7B are a bit wishy-washy. The impressive length of this region might indicate complex interactions with other mitochondrial proteins as proposed here. The analysis of these interactions is certainly beyond the scope of this study. However, the authors should characterize the interactors of the C-terminal membrane region of p166 in more depth. To this end, they might employ their mini-p166-HA and ΔC constructs and subject the immunoprecipitates to mass spectrometry. Given the results shown in Fig. 4B, this should be a straight-forward strategy to characterize the molecular interactions in a more comprehensive and quantitative manner. Such an analysis would be of great interest for others studying the TAC architecture.*

We performed the suggested analyses. The results in the new Fig. S4 shows that TAC60 and TAC40 are the only TAC subunits that get more than 2-fold enriched using mini-p166-HA as a bait, whereas when mini-p166ΔC-HA was used as a bait no TAC subunits were detected. These reciprocal pulldown experiments confirm the results shown in Fig. 5B. Interestingly, the only other proteins that are specifically enriched in the pull down experiment of the mini-p166-HA with the intact C-tail are four subunits of the ATOM complex. This suggests that a fraction of the ATOM complex might be associated with the TAC. The significance of this observation is presently unclear and would require additional analyses which are beyond the scope of the present study.

A paragraph describing the new results has been added to the revised manuscript (page 9, end of first paragraph). The Material and Methods section has been updated accordingly.

*2. The authors identify the C-terminal 39 residues as a specific TAC60 binder. This p166-TAC60 interaction is one of the main conclusions of this study. However, it remains unclear how both proteins interact. According to the alphafold prediction, the IMS region of p166 is unstructured. The interaction assay in yeast shown in Fig. 4C is not very compelling as the p166-HA protein is almost exclusively found in the non-bound fraction. This study would even be stronger if the authors would have characterized the TAC60-p166 interaction in more depth, e.g. by a peptide spot assay or Biacore SPR analysis.*

We agree that the suggested analysis would make the study even stronger. However, a problem we see with the peptide spot assay is that the IMS-exposed loop of TAC60, which we favor as binding site for the p166 C-tail, might not be presented in the correct conformation as a peptide outside of the context of the two flanking transmembrane domain. - Moreover, together with the other additional experiments the 60 days time window for the revision would not have long enough, to properly conduct the suggested experiments considering the lack of experience our lab has in these type of analyses. We therefore hope that the revised version of the manuscript is strong enough to allow for publication without these experiments.

*Reviewer #2: Additional experiments are needed to convincingly show that 1) the predicted TMD is responsible for membrane integration, and 2) the C-tail of p166 is located in the IMS.*

1)In the revised manuscript we now also analyzed a p166 version that lacks the TMD. The resulting protein while still fractionating with the organellar fraction was exclusively recovered in the supernatant in the carbonate extraction (see new Fig. S1B). This indicates that the predicted C-terminal TMD is both necessary and sufficient for membrane integration of p166.

A short paragraph has been added to the manuscript to describe this new result (page 5, end of second paragraph)

2)To confirm the IMS localization of the C-terminus for full length p166 we did a protease protection assay using digitonin-extracted mitoplast fractions. New Fig. 4G shows that the C-terminal HA tag of p166 is removed by proteinase K. Moreover, the integral IM protein mitochondrial carrier protein 5 (MCP5) which has multiple transmembrane domains and the matrix marker mitochondrial heat shock protein (mHsp70) remains intact. However, after solubilization of the IM by Triton-X-100 all proteins are digested. Since p166 contains a single TMD only, these results demonstrate that its C-terminus reaches into the IMS.

A paragraph has been added to the manuscript to describe this new result (page 8, second paragraph)

*Reviewer #3: 1. The MTS. The N-terminal MTS in p166 suggests its presence on IM. Figure 2C using MTS-GFP showed the mitochondrial localization nicely. To confirm the IM presence of the fusion construct and the topology of the protein as shown in Figures 5A and 7, MTS cleavage shall be tested using the MTS-GFP reporter. Presumably, the MTS is cleaved only if the N-terminal region of p166 is imported into the mitochondrial matrix.*

Translocation of the MTS across the inner membrane depends on the membrane potential. Thus disruption of the potential abolishes import of MTS-containing proteins across and into the IM. We have now investigated what happens to the p166 MTS GFP fusion protein after disruption of the membrane potential. The results in new Figure 2E show that after addition of CCCP to the cell culture a slower migrating band accumulates in a time-dependent manner. This indicates, as would be expected, that unprocessed the p166 MTS GFP precursor protein accumulates when translocation of its MTS across the IM is prevented. Thus, we conclude that p166 is an integral IM protein whose import depends on the membrane potential and the presence of a cleavable MTS.

A paragraph has been added to the manuscript to describe this new result (page 6, end of first paragraph).

*2. The C-tail of p166 is not required for TAC localization. While this appeared to be the case for bloodstream form cells (Figure 6), p166-ΔC-HA seemed absent from the TAC in some procyclic cells (Figure 3E is meant to 2E). In the +Tet samples, two cells with duplicated basal bodies were shown. p166-ΔC-HA appeared missing from the new basal bodies in both cells although it was present in cells with just one pair basal bodies but no kinetoplast. Could this be due to delayed assembly of the IM and other modules of the TAC in the absence of full length p166?*

Yes we think this could indeed be due to delayed assembly.

*3. p166 is the only essential integral IM subunit of the TAC (page 9). Perhaps the authors meant to say that p166 is the only essential integral IM subunit identified so far? P166 is convincingly demonstrated to be an essential component, but I am not sure there is enough evidence to support that p166 is the only essential IM subunit and it is sufficient to bridge the distance between the OM and the kinetoplast (as stated in the abstract). To establish sufficiency, think a (semi)-purified system showing that purified p166 alone can link up OM and kintoplast complexes would be required. The current study cannot rule out the presence of other IM or intermembrane subunits required for this bridging, and the data and the model depicted in Figure 7 in fact predicted the involvement of other IM subunit(s).*

We agree with the reviewer and we apologize for this over interpretation of the data. - What we wanted to say is that with all the components known, and with the information of how they interact with each other, the distance between the outer membrane and the kDNA proximal TAC102 can in principle be bridged without gaps. However, this does of course not exclude that further essential TAC subunits may exist.

We have reformulated the phrase in question in the abstract. It now reads: “Whereas the outer membrane region requires four essential subunits for proper TAC function, the inner membrane integral p166, via its interaction with TAC60 and TAC102, would theoretically suffice to bridge the distance between the OM and the kDNA.”

We have also modified the discussion accordingly. The paragraph in question now reads (page 10, second last paragraph): “Presently p166 is the only known essential integral IM subunit of the TAC. Considering that p166 can bridge the gap from the OM to the kDNA, it is possible that p166 is the only IM TAC subunit required for TAC function. However, it is presently not possible to exclude that other as yet unknown IM TAC subunits might exist.”

*Reviewer #4: These results provide important answers to questions about the formation and components of the TAC, and clearly provides new insights, but there are some unanswered questions. I think that this paper is pretty close to be ready for publication in PLoS Path when the corrections below have been adequately addressed.

Major comments

1. Figures 1B, 3D, and 4C, there is no visible statistical robustness in these data (error bars) and this, at least, must be addressed.*

The data showing the kDNA analysis in the indicated figures are very robust. In all analyzed cell lines, we saw either very large effects affecting 50 to 100% of all cells or no difference to the control. These phenotypes are easy to score which is why decided to quantitatively analyze only one representative experiment for each of the tested cell lines. Having said that for all experiments multiple IF slides were analyzed.

*2. Immunoprecipitation using mini-p166-HA as bait would provide some clues as to whether the hypothesis in Fig. 7 is true. What are the proteins identified by IP of mini-p166-HA?*

We performed the suggested analyses. The results in the new Fig. S4 shows that TAC60 and TAC40 are the only TAC subunits that get more than 2-fold enriched using mini-p166-HA as a bait, whereas when mini-p166ΔC-HA was used as a bait no TAC subunits were detected. These reciprocal pulldown experiments confirm the results shown in Fig. 5B. Interestingly, the only other proteins that are specifically enriched in the pull down experiment of the mini-p166-HA with the intact C-tail are four subunits of the ATOM complex. This suggests that a fraction of the ATOM complex might be associated with the TAC. The significance of this observation is presently unclear and would require additional analyses which are beyond the scope of the present study.

Please note we do not think that these pulldown experiments necessarily provide clues regarding the identity of the postulated proteins described in Fig. 7B. The reason is that the mini-p166-HA protein version that lacks the C-tail is not localized to the TAC anymore (Fig. 4), which is in contrast to the exclusively expressed ΔC-tail version of the HA-tagged full length p166 in the L262P bloodstream form cells which still localizes to the TAC (Fig. 6).

A paragraph describing the new results has been added to the revised manuscript (page 9, end of first paragraph). The Material and Methods section has been updated accordingly.

*Reviewer #5: No major issues.*

***Part III – Minor Issues: Editorial and Data Presentation Modifications*** *Please use this section for editorial suggestions as well as relatively minor modifications of existing data that would enhance clarity.*

*Reviewer #1: 3. Fig. 1. Labeling is absent in panel B. Please copy the description from A also to the B panel
4. Fig. 5, legend. (D) should be in bold*

The figure and the legend have been changed accordingly.

*Reviewer #2: Minor points:

- Page 5 line 11; Remove ‘exclusively’. As mentioned by the authors in later section that RNAi may not remove 100% of the wildtype copy.
- Page 5, line 29; replace ‘distant’ by ‘distal’*

Corrected as suggested

*Reviewer #3: Page 7, “less then 3%” shall be “less than 3%”.*

Corrected

*Reviewer #4: Minor comments*

*p166 does not require kDNA to target to the TAC. Where does it enter the mitochondrion and how does it find the TAC? The authors touch upon this in the discussion but is there solid proof that ATOM and/or TIM translocate p166? These are fundamental questions that would help understand how the TAC is built.*

p166 has been detected in the previously determined mitochondrial importome of *T. brucei* (2). The importome includes all proteins that require the OM import pore ATOM40 for import. Moreover, the newly added experiment shown in Fig. 2E shows that p166 has a cleavable N-terminal mitochondrial targeting sequence, since the uncleaved precursor accumulates after disruption of the membrane potential. This is very strong evidence that it requires the single trypanosomal TIM complex for import*.* Thus, from all we know, p166 is a typical substrate of the presequence pathway*.

Figure 1B right panel. After knocking down endogenous p166 the HA tagged version is able to compensate but there are mutant phenotypes present. Please explain the reason for their production.*

While the growth arrest caused by p166 RNAi is completely complemented by expression of p166-HA, a very small percentage (<5%) show a mutant phenotype (please note that in the uncomplemented cell line >95% of cells have a mutant phenotype. The likely explanation for this observation is that in a few cells complementation does not work, possibly because of cell to cell variation in the expression of p166-HA.
 *Figure 1B is not clear. Please label the graphs as WT and p166-HA or something similar and please label all graphs.*

The figures in question were edited as requested.

*Page 4 last sentence, I am not sure what is meant by “After 2 days of induction mitochondria were enriched by digitonin extraction“. I think that there is a word(s) missing between “mitochondria” and “were”.*

We think there is no word missing in this phrase (which is on page 8 and not 4). Nevertheless, we slightly edited the phrase to make it clearer.

*Figure 5 A, the legend does not match the image. There is no dotted box (C-terminal domain) in my image. Also, it may be obvious to some but “inp, FT and IP” need to be described in the legend. Please explain what the 1X and 10 mean on these figures. For 5D, TAC60deltaC-myc is the bait so Please indicate on the figure.*

The Figure and the legends have been changes accordingly.

*Figure 5C right panel. Why does the amount of mini-p166-HA drop in the delta N+C-ter IP bait versus the delta C-ter alone bait if the IMS region of TAC 60 binds to mini-p166-HA ?*

In our opinion there is only marginally less of mini-p166-HA present in the right panel. It is possible that the pulldown of the bait was also slightly less efficient. In addition, the bait construct on the right TAC60ΔNΔC) is not functional (1). It does localize to the TAC region but cannot complement the loss of WT. Hence, some mini-p166-HA might preferentially bind the still existing WT TAC60 in this cell line (there is no RNAi of WT TAC60 in this cell line). Lastly, we state in the manuscript “that the interaction is *at least partially* mediated by the IMS loop of TAC60”.

*Page 7. The sentence; “p166-DC-HA on the other hand is mostly lost in isolated cytoskeleton and flagella possibly because its interaction with the TAC is Triton-X100 sensitive”, does not quite make sense. Should it be more like; p166-DC-HA on the other hand is mostly lost in isolated cytoskeleton and flagella preparations possibly because its interaction with the TAC is Triton-X100 sensitive. Or, p166-DC-HA on the other hand is mostly absent when observing isolated cytoskeletons and flagella possibly because its interaction with the TAC is Triton-X100 sensitive”*

We don’t see much difference of our phrase to the suggested modified phrases. We talk about “isolated cytoskeleton and flagella” to add “preparation” to this phrase seems redundant to us.

*Reviewer #5: The authors state in the abstract, and explore further in the discussion, the idea that p166 is the only TAC subunit in inner membrane. Do the authors mean the only one identified so far or do they think, based on the fairly exhaustive screens that have been performed by this group and others, that all of the core TAC components have been found and that p166 really is the only one in the IM? In contrast to this, the authors also appear to argue that p166, since it localizes to the TAC region in the absence of kDNA and its C-terminal domain, relies on other factors to correctly position it in the differentiated membrane. They even propose in their model that these accessory proteins are probably integral proteins in the IM, and highlight this as an intriguing avenue for future investigation. Wouldn’t these proteins also count as IM TAC components? Do the authors distinguish between “core” and “accessory” components of TAC? This may just be a semantics issue but I think the manuscript would benefit from additional clarity on how the authors are defining true components of TAC versus TAC-interacting/assembly proteins.*

We agree with the reviewer and corrected the paper accordingly for details see above: Part II – Major Issues: Key Experiments Required for Acceptance, Reviewer 3, point 3.

*The abstract states that non-essential TAC-associated proteins in the OM must retain the p166 lacking its C-terminal domain in the TAC region. Perhaps they meant TAC-associated proteins in the matrix or IM perhaps? And how do they know these proteins won’t be essential since they may be necessary for the correct localization of p166?*

We agree we cannot know where these proteins are localized and also not whether they would be essential or not. The abstract has been corrected accordingly.

*Along these lines, the authors point out the interaction between the N-terminal portion of p166 and TAC102. Do they think TAC102 is insufficient to provide the “weak interactions” necessary to retain p166 in the TAC region? In other words, why is it more likely to be an integral membrane protein as opposed to a kDNA binding protein? Hoffmann et al., 2018 seemed to find that p166 localization was disrupted during TAC102 knockdown by RNAi, although some remained. Residual localization of p166 could be is due to incomplete knockdown of TAC102?*

In our opinion Fig. 6 clearly shows that non-functional p166-ΔC-HA can still localize to the TAC in cells that completely lack kDNA (see Fig. 6C and 6D, right most panels). Please note that these are L262P bloodstream form cell lines that can grow in the absence of the kDNA. Moreover, the cell line used is not an RNAi cell line but excusively expresses p166-ΔC-HA, no wildtype gene copy of p166 is present in these cells. These cells therefore lack kDNA and as a consequence have a delocalized p102, thus whatever holds p166-ΔC-HA in place must be “upstream” of p166.

We believe that the original Fig. 6 might have been a bit misleading, as it was not clearly stated with which cell line the experiments were done. In the revised manuscript we have now modified Fig. 6 and the corresponding legend to make it clear which cell line was used for the experiments.

*Bottom of page 6, first paragraph under the heading “The C-tail of p166 reaches into the IMS”. I am convinced by the data in this paper that p166 is in the IM with the topology suggested (N-term in matrix) but is the distance between the two membranes in the differentiated portion really a compelling addition to this argument? Perhaps this could be omitted.*

The argument might not be compelling on its own but it supports the conclusion drawn from the other experiments, we therefore would like to leave it.

*PDF version—arrows in figure 4 left panels seem to be partially obscuring areas of enrichment. Could they be shifted over?*

The figure has been changed accordingly

*Fig 5—maybe it’s just the PDF version again but I don’t see a dotted box for the c-term of TAC60.*

This has been corrected

*Fig 5 IPs—why are there two bands for p166 and why does only one appear to be pulled down?*

As discussed above the anti-HA and anti-myc antibodies are specific. There are no unspecific bands. However, depending on the percentages of the SDS gels used we often see clusters of bands with very similar molecular weights for different TAC subunits. They likely reflect as yet unknown posttranslational modifications. Alternatively or additionally, they could be caused by limited N-terminal proteolysis. – We do not know why only one band appears to be pull down in the experiment.

*Do the authors mention the ATOM40 negative control in the text? Information on bait/target should be indicated on Fig 5D or included in the figure legend. Should also include the information on panel or legend that TOM40 a yeast protein that is serving as a control.*

The legend has been modified accordingly

*In Fig. 5D there are again two bands. I assume p166 is the lower band but why does it appear to run so much smaller than the mini-p166-HA shown in 5B?*

It does not run smaller. The gels were of different percentages and we just show different marker bands.

*In the text for Fig 6 (top of page 9), it was a little unclear what the authors meant by “four cell lines”. Perhaps the diagrams of the p166 locus found in supplemental Fig S3 showing where their primers bind could be moved to the main figure. That would help with interpretation of the gel as well. Alternatively, the text could be altered to make it clear what the four cell lines are.*

Fig. 6 and the corresponding legend have been modified accordingly

***References***

1.	Käser S, Willemin M, Schnarwiler F, Schimanski B, Poveda-Huertes D, Oeljeklaus S, et al. Biogenesis of the mitochondrial DNA inheritance machinery in the mitochondrial outer membrane of Trypanosoma brucei. PLoS Pathog. 2017;13(12):e1006808.

2.	Peikert CD, Mani J, Morgenstern M, Käser S, Knapp B, Wenger C, et al. Charting Organellar Importomes by Quantitative Mass Spectrometry. Nat Commun. 2017;8:15272.
